# Response to Hulliard, Le Strat, Dubertre, et al.

**DOI:** 10.1093/jnci/djz004

**Published:** 2019-01-10

**Authors:** Helena Carreira, Rachael Williams, Martin Müller, Rhea Harewood, Susannah Stanway, Krishnan Bhaskaran

**Affiliations:** 1 Department of Non-Communicable Disease Epidemiology, Faculty of Epidemiology and Population Health, London School of Hygiene & Tropical Medicine, London, UK; 2 Clinical Practice Research Datalink (CPRD) Medicines and Healthcare products Regulatory Agency, London, UK; 3 Department of Emergency Medicine, Inselspital, Bern University Hospital, University of Bern, Bern, Switzerland; 4 Institute of Health Economics and Clinical Epidemiology, University Hospital of Cologne, Cologne, Germany; 5 Department of Medicine, The Royal Marsden NHS Foundation Trust, London and Surrey, UK

Dr Huillard et al. suggested that we report on mental health outcomes in breast cancer survivors with and without history of mental disorders. Twenty-two of the 60 studies excluded participants with history of mental disorders. Of the 38 studies that did not mention psychiatric history in their exclusion criteria, three accounted for it either through matching or adjustment in multivariable analyses; only one study explored the role of psychiatric history (it showed no correlation between psychiatric history and symptoms of posttraumatic stress).

Dr Huillard et al. noted that in a previous study, an increased risk of mental disorders was only observed among cancer patients who had a history of mental disorder (1). As noted above, results stratified by psychiatric history were seldom available in studies that we reviewed. However, we believe that the results of the studies that included only participants with no history of mental disorders are informative. Four population-based studies included in our review, in which outcomes were clinically ascertained, showed an increased risk of anxiety and/or depression in breast cancer survivors with no history of mental disorders, relative to comparable women without cancer (2–5). This shows that for breast cancer survivors (>1 year), the risk of first-ever disorders is increased relative to women who never had cancer. If the hypothesis of Dr Huillard et al. is correct, the burden of mental disorders is likely to be underestimated in the studies restricted to women with no history of mental disorders. We should also note that our study focused solely on female breast cancer survivors at least 1 year after diagnosis, whereas the Huillard et al. study included patients with a wide range of cancers (16% were of the breast). It is plausible that the effect of a cancer diagnosis on the patients’ mental health varies by cancer site. We thank Dr Huillard et al. for their interest in our study.

## Notes

Affiliations of authors: Department of Non-Communicable Disease Epidemiology, Faculty of Epidemiology and Population Health, London School of Hygiene & Tropical Medicine, London, UK (HC, RH, KB); Clinical Practice Research Datalink (CPRD), Medicines and Healthcare products Regulatory Agency, London, UK (RW); Department of Emergency Medicine, Inselspital, Bern University Hospital, University of Bern, Bern, Switzerland (MM); Institute of Health Economics and Clinical Epidemiology, University Hospital of Cologne, Cologne, Germany (MM); Department of Medicine, The Royal Marsden NHS Foundation Trust, London and Surrey, UK (SS).
